# Termination of pregnancy data completeness and feasibility in population-based surveys: EN-INDEPTH study

**DOI:** 10.1186/s12963-020-00238-9

**Published:** 2021-02-08

**Authors:** Yeetey Akpe Kwesi Enuameh, Francis Dzabeng, Hannah Blencowe, Sanne M. Thysen, Solomon Mekonnen Abebe, Kwaku Poku Asante, Charlotte Tawiah, Vladimir Sergeevich Gordeev, Wisdom Adeapena, Doris Kwesiga, Simon Kasasa, Charles Zandoh, Md. Ali Imam, Seeba Amenga-Etego, Sam K. Newton, Seth Owusu-Agyei, Joy E. Lawn, Peter Waiswa, Jenny A. Cresswell, the Every Newborn-INDEPTH Study Collaborative Group, Peter Byass, Stephen M. Tollman, Hagos Godefay, Joy E. Lawn, Peter Waiswa, Hannah Blencowe, Judith Yargawa, Joseph Akuze, Ane B. Fisker, Justiniano S. D. Martins, Amabelia Rodrigues, Sanne M. Thysen, Gashaw Andargie Biks, Solomon Mokonnen Abebe, Tadesse Awoke Ayele, Telake Azale Bisetegn, Tadess Guadu Delele, Kassahun Alemu Gelaye, Bisrat Misganaw Geremew, Lemma Derseh Gezie, Tesfahun Melese, Mezgebu Yitayal Mengistu, Adane Kebede Tesega, Temesgen Azemeraw Yitayew, Simon Kasasa, Edward Galiwango, Collins Gyezaho, Judith Kaija, Dan Kajungu, Tryphena Nareeba, Davis Natukwatsa, Valerie Tusubira, Yeetey A. K. Enuameh, Kwaku P. Asante, Francis Dzabeng, Seeba Amenga Etego, Alexander A. Manu, Grace Manu, Obed Ernest Nettey, Sam K. Newton, Seth Owusu-Agyei, Charlotte Tawiah, Charles Zandoh, Nurul Alam, Nafisa Delwar, M. Moinuddin Haider, Md. Ali Imam, Kaiser Mahmud, Angela Baschieri, Simon Cousens, Vladimir S. Gordeev, Victoria Ponce Hardy, Doris Kwesiga, Kazuyo Machiyama

**Affiliations:** 1 Kintampo Health Research Centre, Kintampo, Ghana; 2 Dept of Epidemiology & Biostatistics, Kwame Nkrumah University of Science & Technology, Kumasi, Ghana; 3 Maternal, Adolescent, Reproductive & Child Health (MARCH) Centre, London Sch. of Hygiene & Tropical Medicine, London, UK; 4 Bandim Health Project, Bissau, Guinea-Bissau; 5 Research Centre for Vitamins and Vaccines, Statens Serum Institut, Copenhagen, Denmark; 6 Department of Clinical Research Open Patient data Explorative Network (OPEN), University of Southern Denmark, Odense, Denmark; 7 Dabat Research Centre Health and Demographic Surveillance System, Dabat, Ethiopia; 8 Department of Health Systems and Policy, Institute of Public Health, University of Gondar, Gondar, Ethiopia; 9 The Institute of Population Health Sciences, Queen Mary University of London, London, UK; 10 Department of Health Policy, Planning and Management, Makerere University School of Public Health, Kampala, Uganda; 11 International Maternal & Child Health, Department of Women and Children’s Health, Uppsala University, Uppsala, Sweden; 12 Centre of Excellence for Maternal Newborn and Child Health Research, Makerere University, Kampala, Uganda; 13 IgangaMayuge Health and Demographic Surveillance System, Iganga, Uganda; 14 Makerere University Centre for Health and Population Research, Makerere, Uganda; 15 Department of Epidemiology & Biostatistics, Makerere University School of Public Health, Kampala, Uganda; 16 Health Systems and Population Studies Division, icddr,b, Dhaka, Bangladesh; 17 Department of Global Health, Kwame Nkrumah University of Science & Technology, Kumasi, Ghana; 18 Institute of Health Research, University of Health and Allied Sciences, Ho, Ghana; 19 Centre of Excellence for Maternal Newborn and Child Health Research, Makerere University, Kampala, Uganda; 20 Department of Global Public Health, Karolinska Institutet, Stockholm, Sweden

**Keywords:** Population-based surveys, Household survey, Completeness, Termination of pregnancy, Abortion, Health and demographic surveillance

## Abstract

**Background:**

Termination of pregnancy (TOP) is a common cause of maternal morbidity and mortality in low- and middle-income countries. Population-based surveys are the major data source for TOP data in LMICs but are known to have shortcomings that require improving. The EN-INDEPTH multi-country survey employed a full pregnancy history approach with roster and new questions on TOP and Menstrual Restoration. This mixed methods paper assesses the completeness of responses to questions eliciting TOP information from respondents and reports on practices, barriers, and facilitators to TOP reporting.

**Methods:**

The EN-INDEPTH study was a population-based cross-sectional study. The Full Pregnancy History arm of the study surveyed 34,371 women of reproductive age between 2017 and 2018 in five Health and Demographic Surveillance System (HDSS) sites of the INDEPTH network: Bandim, Guinea-Bissau; Dabat, Ethiopia; IgangaMayuge, Uganda; Kintampo, Ghana; and Matlab, Bangladesh. Completeness and time spent in answering TOP questions were evaluated using simple tabulations and summary statistics. Exact binomial 95% confidence intervals were computed for TOP rates and ratios. Twenty-eight (28) focus group discussions were undertaken and analysed thematically.

**Results:**

Completeness of responses regarding TOP was between 90.3 and 100.0% for all question types. The new questions elicited between 2.0% (1.0–3.4), 15.5% (13.9–17.3), and 11.5% (8.8–14.7) lifetime TOP cases over the roster questions from Dabat, Ethiopia; Matlab, Bangladesh; and Kintampo, Ghana, respectively. The median response time on the roster TOP questions was below 1.3 minutes in all sites. Qualitative results revealed that TOP was frequently stigmatised and perceived as immoral, inhumane, and shameful. Hence, it was kept secret rendering it difficult and uncomfortable to report. Miscarriages were perceived to be natural, being easier to report than TOP. Interviewer techniques, which were perceived to facilitate TOP disclosure, included cultural competence, knowledge of contextually appropriate terms for TOP, adaptation to interviewee’s individual circumstances, being non-judgmental, speaking a common language, and providing detailed informed consent.

**Conclusions:**

Survey roster questions may under-represent true TOP rates, since the new questions elicited responses from women who had not disclosed TOP in the roster questions. Further research is recommended particularly into standardised training and approaches to improving interview context and techniques to facilitate TOP reporting in surveys.

## Key findings

**Table:** 

**WHAT IS NEW?**
• **What was known already:** Termination of pregnancy (TOP), especially if unsafe, remains a significant cause of maternal death, especially in low-and middle-income countries. Population-based surveys are key, yet are known to under-capture TOP. • **What was done:** The EN-INDEPTH multi-country survey included a full pregnancy history by roster and new questions on TOP and Menstrual Restoration. Data on 34,371 women were analysed to assess the completeness of responses to roster and new questions to elicit TOP information. Perceptions, practices, barriers, and facilitators to TOP reporting were studied.
**What was found in the quantitative data?**
• **Completeness of responses:** Was high for roster, new TOP questions, and Menstrual Restoration questions and ranged between 90.3 and 100%. The median response time on the roster TOP questions was below 1.3 minutes in all sites. • **Data utility:** When the new TOP questions were used, between 2.0 and 15.5% of women who had not disclosed TOP in the roster questions reported TOP. The highest proportion of women who reported TOP in the Roster (14.0 %) and new TOP questions (15.5%) were from Matlab, Bangladesh. IgangaMayuge, Uganda, had the highest proportion who reported having used Menstrual Restoration (17.2 %). Termination of pregnancy rates for the 5 years preceding the EN-INDEPTH survey generated from the Full Pregnancy History roster questions ranged between 0.3 (Dabat, Ethiopia) to 19.3 (Kintampo, Ghana) TOPs per 1000 women aged 15–49 years.
**What was found in the qualitative data?**
• **Barriers/enablers to reporting** ○ Termination of pregnancy was perceived as difficult and uncomfortable to disclose by many respondents. This was due to its perceived immoral, inhumane, or shameful nature that made it a secret to be kept by women. Miscarriages felt natural so were perceived as easier to disclose than TOP by women. ○ Good interviewer techniques including cultural competence, knowledge of contextually appropriate expressions of TOP, adaptation to interviewee’s individual circumstances, being non-judgmental, speaking a common language, and providing detailed informed consent were perceived by interviewers and women to facilitate TOP disclosure.
**What next in measurement and research?**
• **Measurement improvement now:** Interviewers for future population-based surveys should be selected and given standardised training with enhanced appreciation of interview techniques specifically to address stigma and contextual factors to facilitate TOP reporting. Improving survey processes, including use of non-judgemental language in translations of questions and prompts, is necessary. • **Research needed:** Further adequately powered experimental studies are needed to validate the use of new TOP questions in eliciting information on TOP to improve monitoring of this outcome in surveys.

## Background

Accurate termination of pregnancy (TOP) rates are difficult to obtain, particularly in low- and middle-income countries (LMICs) with restrictive laws, or where incomplete coverage of routine data collection systems requires reliance on household surveys where under-reporting is common. Globally, some reduction has been seen in TOP rates from an estimated 40 per 1000 females aged 15–49 years in 1990–1994, to 35 per 1000 in 2010–2014 [1, 2]. However, whilst TOP rates have declined significantly in high-income countries (HICs), they have remained roughly constant in LMICs [1]. Current rates are 36 and 27 per 1000 for LMICs and HICs, respectively [1]. Unsafe TOPs are significantly higher in LMICs with highly restrictive TOP laws compared to HICs with less restrictive laws [3–5]. Up to 55.7 million TOPs occurred each year between 2010 and 2014 worldwide, of which 25.1 million (45.1%) were unsafe; LMICs contributed 24·3 million (97%) of these [4]. Unsafe TOPs contribute significantly to maternal morbidity and mortality [3, 5, 6]. However, underreporting of TOP remains a universal concern as evidenced in some countries with relatively liberal laws in which between 20 and 60% of cases were not disclosed during surveys [7–10].

Data regarding TOP in LMICs is predominantly generated from health facility data and household surveys, notably Demographic and Health Surveys (DHS), plus special Maternal Health Surveys [11]. Other studies have employed different methods to collect information on TOP such as the confidante method, the list experiment, the abortion incidence method (AICM), and the modified AICM [12–14].

DHS surveys have, until now, generally employed standard questionnaires with a Full Birth History approach (FBH) to collect information on pregnancy losses, not distinguishing between induced (TOP) and spontaneous abortions (miscarriages) [15–17]. The Full Pregnancy History (FPH), however, elicits details of all pregnancy outcomes, facilitating distinctions between TOP and miscarriages [15]. Termination of pregnancy data collected via face-to-face DHS have known limitations including misreporting and underreporting, which may be due to recall bias or provision of socially desirable responses, amongst others [18, 19]. Previous studies have found that interviewers may influence TOP reporting outcomes [20] and perceived stigma led to misreporting of TOP as miscarriages in surveys in some communities [21].

Termination of pregnancy rates are known to be related to fertility levels of populations and access to TOP. The total fertility rates for the sites included in the EN-INDEPTH study were Matlab, Bangladesh—2.6; Dabat, Ethiopia—3.8; Kintampo, Ghana—4.1; IgangaMayuge, Uganda—4.3; and Bandim, Guinea-Bissau—4.2 (urban) and 5.1 (rural) [15]. All countries involved in this study are considered as having restrictive TOP laws [3, 22, 23]. Previously reported TOP rates for Bangladesh [24, 25], Ethiopia [26], Ghana [12], and Uganda [27] were 29, 28, 44, and 39 per 1000 women aged 15–49 years, respectively. No studies were found reporting TOP rates in Guinea-Bissau.

Few studies have assessed the performance of survey questions by virtue of their completeness of responses in the capture of TOP data in LMICs. Sedgh et al. observed the challenge in generating valid and reliable abortion statistics, recommending additional research to improve monitoring of trends [2]. The extent to which study participants fully respond to key questions related to TOP is referred to as “data completeness” in the context of this manuscript.

This paper is part of a series of papers from the Every Newborn-International Network for the Demographic Evaluation of Populations and their Health (EN-INDEPTH) study in five Health and Demographic Surveillance System (HDSS) sites in Africa and Asia, to improve the measurement of pregnancy outcomes in population-based household surveys. This paper addresses the following objectives:

1. ***Quantitative analysis of performance of TOP survey questions:*** To evaluate modified and new questions’ influence on TOP reporting by women in a population-based survey, including completeness, time spent responding to questions, and plausibility of reported TOP rates.
2. ***Qualitative assessment regarding TOP reporting***: To describe community perceptions, practices, barriers, and facilitators to reporting TOP from the perspective of interviewers and mothers, and how these influenced TOP capture in the EN-INDEPTH survey.

## Methods

### Study design and setting

The EN-INDEPTH study was a population-based cross-sectional study undertaken between July 2017 and August 2018. Five HDSS sites belonging to the INDEPTH Network were involved in the study. The sites are located in Dabat, Ethiopia; Bandim, Guinea-Bissau; IgangaMayuge, Uganda; Kintampo, Ghana; and Matlab, Bangladesh. The study’s primary objective was to compare two methods of retrospective recording of pregnancy outcomes used in the DHS, i.e. a Full Birth History with additional questions on pregnancy losses (FBH+), as per the DHS7 standard and the 2016 Nepal DHS Full Pregnancy History (FPH). Whilst some sites were familiar with FBH+, no sites had previous experience of FPH. To contribute to the understanding of the measurement of TOP in population-based surveys, focus group discussions (FGDs) with women survey respondents and interviewers (Additional file 1) were performed between February and August 2018 [28]. Information on perceptions, practices, barriers, and facilitators to reporting TOP and understanding its measurement was collected.

### Study population and sample

Women aged 15–49 years totalling 69,176 participated in the EN-INDEPTH survey. Survey questions on FBH+ and FPH were administered to 34,805 and 34,371 women, respectively, and data were collected on Android tablets using Survey Solutions software [29]. The study protocol provides details on site selection, sampling processes for the modules, and other related information [15]. Findings of the primary objectives of the study have been published elsewhere [28, 30].

### Participant selection and training

The quantitative arm of the study had women participants from the five HDSS sites being randomly assigned individually to either the FBH+ or the FPH [15, 30]. Women participants of the EN-INDEPTH survey and interviewers who conducted the survey were purposively sampled for the qualitative arm of the study [15, 28]. Contextual factors such as religion and availability of skilled personnel amongst others could have influenced the choice of interviewers for the survey.

Training of the data collectors for both the qualitative and quantitative aspects of this study was based on manuals adapted from the standard DHS interviewer manual [28] and those of the World Bank Survey Solution manuals [15]. The training was broad in nature covering all questions related to the survey and focused group discussions.

### EN-INDEPTH survey questions on TOP in the FPH questionnaire

All women in the FPH arm of the EN-INDEPTH study provided information on all pregnancies in their lifetime regardless of the outcome. Menstrual Restoration questions and additional new TOP questions sought to further explore pregnancy outcomes that were not reported by the women in the roster section. Four sets of questions related to TOP were used: (i) standard TOP questions from the FPH roster were used to generate TOP rates/ratios for all five HDSS sites in the 5 years preceding the survey (Additional files 2 and 3); (ii) a single roster question assessed whether pregnancies earlier reported as “born dead” or “lost before term” were spontaneous or induced losses for all five HDSS sites (Additional file 4); (iii) new questions on Menstrual Restoration elicited information on measures respondents employed for resumption of missed periods for Dabat, Ethiopia; IgangaMayuge, Uganda; and Kintampo, Ghana; (iv) new questions on TOP probed respondents on the standard TOP questions who hitherto did not report TOP in Dabat, Ethiopia; Kintampo, Ghana; and Matlab, Bangladesh. These questions enquired of any unwanted pregnancies that ended up in non-live pregnancy outcomes (Additional file 4).

### Data analysis

#### Objective 1: Quantitative analysis of performance of TOP survey questions

We computed point estimates and exact binomial 95% confidence intervals for TOP rates and ratios (Additional files 2 and 3). Completeness of responses was assessed for the FPH roster questions, and new questions on Menstrual Restoration and TOP in percentages—as number of responses to a question (numerator) per all eligible responses (denominator) to that question. “Yes” and “No” were the response options to the completeness questions, “Don’t know” was not an option (Additional file 4). The essence of this analysis was to explore how feasible it is for women from sites with restrictive TOP laws to respond to such sensitive questions.

Paradata gathered by the Survey Solutions software [29] provided information on time spent responding to questions in all three sets of TOP questions. The mean and median times taken to answer all TOP questions were assessed at each of the five HDSS sites. Descriptive analyses were done to compare women’s response time to TOP questions by HDSS sites. To account for the variation in number of TOP questions asked to women by HDSS, we standardised the time taken by the total number of TOP questions asked by each HDSS site. During analysis, the time was truncated to 0.5–30 min to eliminate outliers due to interruptions of interviews. Shorter response times documented by paradata were to signify less time spent on questions and by inference less time burden on respondents. Longer times could however imply respondents struggling with understanding of questions or interviewers having issues with their administration [31]. Stata version 15.1 was used for quantitative data management and analyses [32].

Results are reported in accordance with STROBE Statement checklist for cross-sectional studies [22] (Additional file 5).

#### Objective 2: Qualitative assessment regarding TOP reporting

Thematic analysis was conducted using an iterative process guided by an a priori codebook and addition of new codes that emerged during analysis [28]. Themes were summarised and grouped to explore how findings influence the measurement of TOP in population-based surveys. Qualitative data were transcribed using a combination of notes and audio recordings that were coded and analysed using NViVo version 12 [33].

## Results

**Fig. 1:**
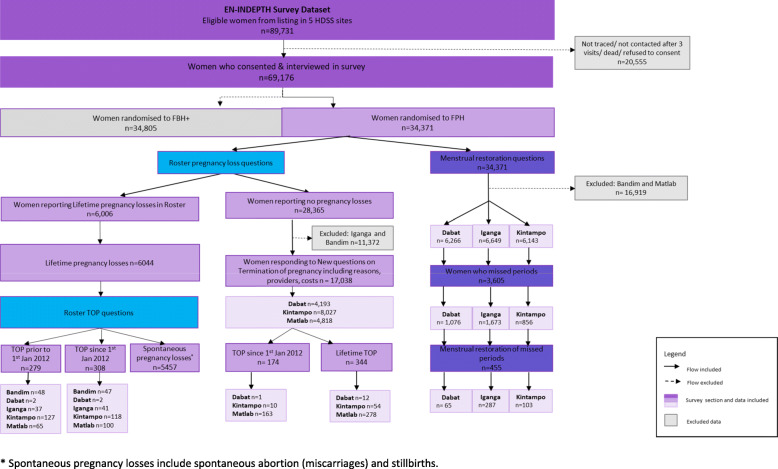
Flow chart of key components of data collection and analysis

Information on TOP was collected from a sample of 34,371 women of reproductive age who participated in the FPH arm of the EN-INDEPTH survey (Fig. 1).

For the quantitative arm of the study, age distribution varied by site and ranged between 15 and 49 years. Respondents with no education were mostly in Dabat, Ethiopia, and Kintampo, Ghana, and those with higher levels were in IgangaMayuge, Uganda, and Matlab, Bangladesh, with Bandim, Guinea-Bissau being mid-way between the two groups. Majority in IgangaMayuge, Uganda, and Matlab, Bangladesh, were Muslim with Christianity dominating in the other three sites. Parity was predominantly three and below except for Kintampo, Ghana (Table 1).

**Table 1:** Socio-demographic characteristics of EN-INDEPTH survey FPH group respondents

Health and demographic surveillance system sites						
	Bandim, Guinea-Bissau (***N*** = 4660)	Dabat, Ethiopia (***N*** = 6266)	IgangaMayuge, Uganda (***N*** = 6649)	Matlab, Bangladesh (***N*** = 10,653)	Kintampo, Ghana (***N*** = 6143)	Total (***N*** = 34,371)
	*n* (%)	*n* (%)	*n* (%)	*n* (%)	*n* (%)	*n* (%)
**Age**						
15–19	220 (4.7)	1337 (21.3)	1912 (28.8)	547 (5.1)	149 (2.4)	4165 (12.1)
20–24	1037 (22.3)	961 (15.3)	1319 (19.8)	2722 (25.6)	903 (14.7)	6942 (20.2)
25–29	1207 (25.9)	1053 (16.8)	896 (13.5)	3067 (28.8)	1285 (20.9)	7508 (21.8)
30–34	1072 (23.0)	857 (13.7)	686 (10.3)	2554 (24)	1499 (24.4)	6668 (19.4)
35+	1116 (23.9)	2058 (32.8)	1831 (27.5)	1763 (16.5)	2301 (37.5)	9069 (26.4)
Missing	8 (0.2)	0 (0.0)	5 (0.1)	0 (0.0)	6 (0.1)	19 (0.1)
**Education level**						
No education	1291 (27.7)	3047 (48.6)	444 (6.7)	487 (4.6)	2418 (39.4)	7687 (22.4)
Primary only	1366 (29.3)	1688 (26.9)	3018 (45.4)	2017 (18.9)	2754 (44.8)	10,843 (31.5)
Primary and secondary	1656 (35.5)	877 (14)	2763 (41.6)	6690 (62.8)	892 (14.5)	12,878 (37.5)
Higher	340 (7.3)	654 (10.4)	420 (6.3)	1457 (13.7)	75 (1.20)	2946 (8.6)
Missing	7 (0.2)	0 (0.0)	4 (0.1)	2 (0.0)	4 (0.1)	17 (0.1)
**Religion**						
Christian	1907 (41)	6009 (95.9)	3039 (45.8)	0 (0.0)	3875 (63.1)	14,830 (43.2)
Muslim	1834 (39.4)	257 (4.1)	3592 (54.1)	9430 (88.5)	1889 (30.8)	17,002 (49.5)
Other or none	908 (19.5)	0 (0)	11 (0.2)	1221 (11.5)	374 (6.1)	2514 (7.3)
**Parity**						
3 or less births	3099 (66.5)	3815 (60.9)	4208 (63.3)	9361 (87.9)	3073 (50.0)	23,556 (68.5)
> 3 births	1561 (33.5)	2451 (39.1)	2441 (36.7)	1292 (12.1)	3070 (50.0)	10,815 (31.5)
**Socioeconomic status**						
Poorest	938 (20.1)	1427 (22.8)	1321 (19.9)	2175 (20.4)	1226 (20.0)	7087 (20.6)
2	893 (19.2)	1074 (17.1)	1319 (19.8)	2074 (19.5)	1248 (20.3)	6608 (19.2)
3	964 (20.7)	1244 (19.9)	1342 (20.2)	2125 (19.9)	1273 (20.7)	6948 (20.2)
4	969 (20.8)	1255 (20.0)	1358 (20.4)	2133 (20.0)	1211 (19.7)	6926 (20.2)
Richest	896 (19.2)	1266 (20.2)	1309 (19.7)	2146 (20.1)	1185 (19.3)	6802 (19.8)

For the qualitative arm of the study, two-thirds of women and a third of interviewers were aged between 25 and 34 years. Female interviewers were the majority in Bandim, Guinea Bissau; Dabat, Ethiopia; and Matlab, Bangladesh, the minority in Kintampo, Ghana, and evenly split in IgangaMayuge, Uganda (Additional file 6).

### Quantitative analysis of performance of TOP survey questions

Completeness of responses to the roster TOP question was 99.7% (Table 2). Lifetime TOP rates generated from the roster TOP questions were as follows: 13.2% (11.4–15.2) (Kintampo, Ghana), 14.0% (9.0–15.7) (Matlab, Bangladesh), 6.8% (5.5–8.2) (Bandim, Guinea-Bissau), 6.0% (4.8–7.5) (IgangaMayuge, Uganda), and 1.2% (0.3–2.9) (Dabat, Ethiopia) (Table 3). Furthermore, 5-year TOP ratios prior to the EN-INDEPTH survey for all sites ranged from 0.4 (0.0–1.4) to 12.5 (10.3–14.9) per 1000 livebirths (Additional file 3) and TOP rates from 0.3 (0.0–1.2) to 19.2 (15.9–22.13) per 1000 women aged 15–49 years (Additional file 2).

**Table 2:** Completeness of responses to questions regarding termination of pregnancy in the EN-INDEPTH survey

Index question	No. of responses made	Base question	No. of expected responses	Completeness of responses (%)	Comments
a. **Roster TOP questions** (Bandim, Dabat, Iganga-Mayuge, Kintampo, Matlab)—Directly eliciting information as to whether pregnancies of babies born dead or lost before birth had been tampered with					
Did you or someone else do something to end this pregnancy? (**yes/no**)	6044	RQ1: Was the “rostertitle” born alive, born dead, or lost before birth? (alive/**dead/lost before term**)	6064	6044/6064 = (99.7%)	The base question focused on those born “dead or lost before term”
b. **New Menstrual Restoration questions** (Dabat, Iganga-Mayuge, and Kintampo)—Eliciting information as to whether those who had missed their period did something for it to resume, how and where that was done					
Did you do anything to resume your period? (**yes/no**)	3605	AQ1: Have you ever had a period that was more than one week late? (**yes**/no)	3605	3605/3605 = 100.0%	Those with “yes” responses in the base question, answered the index questions over the next three questions.
What did you do to resume your period? (**pill/injectables/herb/other**)	455	AQ2: Did you do anything to resume your period? (**yes**/no)	455	455/455 = 100.0%	
Where did you go to get help to get your period back? (**venue**)	411	AQ2: Did you do anything to resume your period? (**yes**/no)	455	411/455 = 90.3%	
c. **NEW TOP questions** (Dabat, Kintampo, and Matlab)—focused on those who did not report a TOP at the roster level, asking them of unwanted pregnancies that did not result in birth of a live child. It then further probes the outcome of the non-live birth.					
It is not uncommon for a woman to get pregnant at a time when circumstance would make it difficult to have a child. Have you ever gotten pregnant at a time when it would have been difficult for you to have a child, or when you did not want to have one? (***yes*/*no***)	17038	NQ1: Women sometimes have pregnancies that do not result in a live born child. That is, a pregnancy can end in a miscarriage, abortion or the child can be born dead. Have you ever had a pregnancy that did not end in a livebirth? (yes/***no***)	17,071	17,038/17,071 = 99.8%	Those who answered “no” to the base question are those who responded to the index question
Did you or anyone else ever successfully do anything to end that pregnancy? (**yes/no**) [abortion over lifetime]	2870	NQ2: It is not uncommon for a woman to get pregnant at a time when circumstance would make it difficult to have a child. Have you ever gotten pregnant at a time when it would have been difficult for you to have a child, or when you did not want to have one? (***yes***/no)	2870	2870/2870 = 100.0%	Those who responded “yes” to the base questions were those responding to the index question.
Did you have such a pregnancy in the last 5 years? (**yes/no**)	344	NQ3: Did you or anyone else ever successfully do anything to end that pregnancy? (***yes***/no) [abortion over lifetime]	344	344/344 = 100.0%	Response is count of pregnancies
What was the **MAIN reason** you decided to have this (last) abortion?	174	NQ4: Did you have such a pregnancy in the last 5 years? (***yes***/No)	174	174/174 = 100.0%	Response is count of reasons
What **did you do** to end this pregnancy?	174	NQ4: Did you have such a pregnancy in the last 5 years? (***yes***/no)	174	174/174 = 100.0%	Response is count of action taken
**Who suggested** that you might have an abortion?	174	NQ4: Did you have such a pregnancy in the last 5 years? (***yes***/no)	174	174/174 = 100.0%	Response is count of persons who made the suggestion
**Who did you see** to get this done?	174	NQ4: Did you have such a pregnancy in the last 5 years? (***yes***/no)	174	174/174 = 100.0%	Response is count of persons seen
**Where did you go** to get this done?	174	NQ4: Did you have such a pregnancy in the last 5 years? (***yes***/no)	174	174/174 = 100.0%	Response is count of places they went
**How much did you pay** for this abortion, including gifts or money given to the doctor (or person who performed this abortion)?	174	NQ4: Did you have such a pregnancy in the last 5 years? (***yes***/no)	174	174/174 = 100.0%	Response is counts of payments made

**Table 3:** Percent of women reporting lifetime events^a^ in roster, new Menstrual Restoration, and TOP questions of EN-INDEPTH Survey

Site	Roster TOP question	New menstrual restoration questions	New TOP questions
**Percent (95% confidence interval [CI])**			
Bandim	6.7 (5.5–8.2)	–	–
Dabat	1. 2 (0.3–2.9)	6.0 (4.7–7.6)	2.0 (1.0–3.4)
IgangaMayuge	6.0 (4.8–7.5)	17.2 (15.4–19.0)	–
Kintampo	13.2 (11.4–15.2)	12.0 (9.9–14.40)	11. 5 (8.8–14. 7)
Matlab	14.0 (12.4–15.7)	–	15.5 (13.9–17.3)

^a^Events are TOP, restoration of menstruation and TOP, respectively

Completeness of responses to questions was high: on menstrual restoration (i.e. having done something to restore missed periods)—100.0%; on methods employed—100.0%; and place where it was restored—90.3% (Table 2). The lifetime percentage of women who reported having their menstruation periods restored was 17.2% (15.4–19.0) (IgangaMayuge, Uganda), 12.0% (9.9–14.4) (Kintampo, Ghana), and 6.0% (4.7–7.6) (Dabat, Ethiopia) (Table 3).

Completeness of responses was 99.8% to the question on actions being successfully taken to end unwanted pregnancies, and 100.0% for questions on steps taken to terminate unwanted pregnancies, terminations that occurred in the past 5 years, main reasons for the termination, how the termination was done, person who suggested the termination, who performed the termination, where the termination was done and the cost of the termination process (Table 2). The new questions on TOP elicited an extra 2.0% (1.0–3.4), 15.5% (13.9–17.3), and 11.5% (8.8–14. 7) lifetime TOP cases over the roster TOP questions from Dabat, Ethiopia; Matlab, Bangladesh; and Kintampo, Ghana, respectively (Table 3 and Additional file 7). Furthermore, 8.3% (0.2–38.5), 58.6% (52.6–64.5), and 18.5% (9.3–31.4) of the lifetime TOPs in Dabat, Ethiopia; Matlab, Bangladesh; and Kintampo Ghana, respectively, occurred in the last 5 years prior to the EN-INDEPTH survey (Additional file 7). It should be emphasised that these respondents had hitherto not reported having had a TOP from the roster TOP questions.

**Fig. 2:**
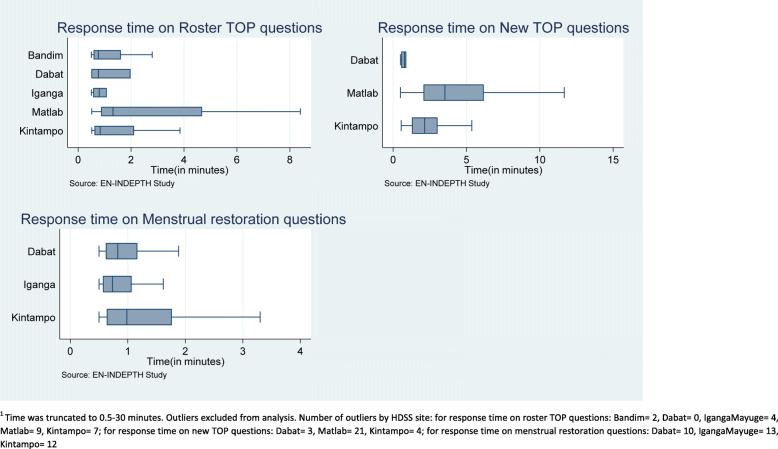
Box plot of response time to complete TOP section in the FPH by HDSS site

The median response time on the roster TOP questions was below 1.3 minutes in all sites. Mean times of responses to roster questions was relatively low in Dabat (1.1 ± 0.8 min) and high in Matlab (3.7 ± 4.7 min) (Fig. 2 and Additional file 8). The mean response time of women to new questions on TOP in Dabat, Ethiopia, was lower (1.5 ± 1.9) than that of Matlab, Bangladesh, (5.0 ± 4.8) and Kintampo, Ghana (2.5 ± 2.2). In addition, women in three HDSS sites (Dabat, IgangaMayuge, and Kintampo) spent less than 2 min on average in responding to the menstrual restoration questions (Fig. 2 and Additional file 8).

### Qualitative assessment regarding TOP reporting

Findings from the qualitative arm of the study are presented under the themes perceptions, practices, barriers, and facilitators to reporting of TOP (Table 4).

**Table 4:** Perceptions, practices, barriers, and facilitators to reporting termination of pregnancy in five EN-INDEPTH study sites

	Woman	Interviewer
**Perceptions on reporting TOPs**		
TOPs immoral and inhuman	- Enquiring of a bad event is perceived unethical (Dabat, Ethiopia) - TOP perceived as taking a life *Why did you abort the baby? Why did you kill a life? What would have been the problem if you had that child? Can’t you rear it up? If you think you cannot take care of it then you have [to] give it to someone for adoption! But why did you abort the baby?* (Iganga-Mayuge, Uganda)	- Culture and religion consider TOP as immoral and inhuman *Also, in the Christian community, abortion is seen to be a bad thing, so it is very difficult to get responses to questions on abortion* (Kintampo, Ghana)
**Perceived TOP reporting practices**		
Difficulty reporting TOPs	- It is one’s secret and shameful to tell others *That one (abortion) is a secret so you won’t disclose it to people apart from telling your trusted friend. Maybe you didn’t know what to do and it was that friend who advised you on how to carry out the abortion. So, if you tell someone about it, the day the two of you will have arguments, the person will use it to insult you. It is shameful to tell people that you have aborted a pregnancy, so it is not possible to easily disclose it* (Kintampo, Ghana) - Pain of loss perceived to be self-afflicted, so one cannot openly grieve *In that case (TOP) there is no grievance. If she actually felt bad, then does she ever do such thing?…If I have to do that unwillingly, I will have to bear more sorrow* (Matlab, Bangladesh)	- It is much more comfortable reporting livebirths. *If you ask the woman she does not want to talk about abortion, she only wants to talk about those children that she gave birth to* (Bandim, Guinea-Bissau) - Interviewers not being empathetic and patient *Sensitive questions must be asked systematically and with care so as not to remind the women of their loss. Being empathetic and patient with her can yield the right information. The problem is most data collectors are in a hurry and not conscious of the women’s grief, so they to ask directly about this sensitive issue. Consequently, the interview ends up with the wrong information* (Dabat, Ethiopia) - Young girls generally shy away from reporting TOPs *Then the other thing on the part of the girls, most of them were not open to disclose information about pregnancies. Some of them were shying away from telling us that they aborted…*(Iganga-Mayuge, Uganda) - TOPs reported as miscarriages instead *We are realizing this challenge of abortion that some women can’t tell you that this abortion was intended. This is a big problem to me. She can’t tell you that this was a miscarriage but when you reach here, she says no, so they hide the information. She says it was a miscarriage, but she fears to tell you that this was intended abortion* (Iganga-Mayuge, Uganda)
**Perceived barriers to reporting TOPs**		
Interviewer unable to ask TOP questions or perceived as disrespectful	- It is a taboo to ask a woman about TOP *If there is an abortion or other adverse events, another pregnancy will come soon as the woman is not lactating. Enquiring about such adverse events is really miserable, perceived as a taboo and as wanting to harm the woman* (Dabat, Ethiopia)	- Interviewers who probe on TOP are perceived as disrespectful *The difficulty is when you ask a woman something, and she says, “is this what you are asking me, my son?” I say, “No auntie, ok, ok, we just want to know how many children, it is not like this, but we want to know how many abortions you have had”. If you don’t ask her in this way, the person becomes awkward and thinks you lack respect (lebsimente) because you seem keen to know about their life* (Bandim, Guinea-Bissau) - Interviewer’s fear to ask direct questions on TOP due to legal and religious reasons *There is a challenge though somehow not that serious that relates to issues of legal and religion…we hear that abortion is illegal and when it comes to the question that asks direct that “did you do anything to end this pregnancy?” this it requires you to be trained and less judgmental* (Iganga-Mayuge, Uganda)
Stigma associated with TOPs	- TOPs are perceived to be linked to heredity and fear reoccurrence of the problem in the next pregnancy (Dabat, Ethiopia)	- Younger females not willing to disclose TOPs *Some of them were shying away from telling us that they aborted* (Iganga-Mauyge, Uganda) - Persons who have done TOPs are stigmatised and perceived as murderers. *I think the perception the community has about abortion…how they stigmatize those who have done abortions... Sometimes you are seen as a murderer, so someone who has had an abortion would not like to open up* (Kintampo, Ghana) - Miscarriages easier reporting than TOPs as they are perceived as natural *For me personally getting information on miscarriages is not as difficult as that of abortion because they see the miscarriage as a natural occurrence* (Kintampo, Ghana)
Very uncomfortable reporting TOPs	- Lots of women did not want to think about, more so talk about TOPs *God gave and God took it!* (Dabat, Ethiopia)	- Some women are just uncomfortable disclosing TOPs *Some of them feel very uncomfortable giving you such details and also some of them find it difficult in giving you details about children that they have lost depending on the circumstance under which they lost the child* (Kintampo, Ghana)
Poor documentation of TOPs		- There are rarely records of TOPs as compared to livebirths, etc. so information solely depends on what the woman provides *There is a bit of records to get on the neonatal births and livebirths as compared to miscarriages and abortion. It also depends solely on the woman to give you all the details of since there are no records on it in the ANC so it all boils down to the kind of rapport and relationship you build with her* (Kintampo, Ghana)
**Perceived facilitators of TOP reporting**		
Good interviewer techniques		- Making use of “expressions” that tacitly inquire about TOPs in place of explicit language *What makes it easier for us is instead of asking the woman how many times she has had an abortion; you ask her how many times she has ‘taken out her stomach’* (Bandim, Guinea-Bissau) - Communicating with the husband or her mother is one way of getting reliable information (Dabat, Ethiopia) - Or speaking to the woman after she recovers from her grief may be another alternative. Interviewer must be able to gauge the right moment to ask questions *You have to also be careful not to directly go to the questionnaire. You must first get her attention and concentration by understanding her feelings, facial expressions, and readiness to share the information* (Dabat, Ethiopia) - TOPs are stigmatised and reasons for carrying them out are diverse, so interviewer’s craftiness facilitates disclosure *With the abortions, we have made it look like something that is really bad and scary. If someone has committed abortion, she is even afraid of saying it because she thinks she will be reported for the society to have a certain perception about her. There are different reasons why someone will have an abortion, some young girls are afraid of their parents so when they get pregnant, they will rather choose to abort it, some are also based on doctors’ advice. So, you have to be really careful when asking on the field* (Kintampo, Ghana)
A common language of interaction		- Speaking the language of the interviewee enhance disclosure *Apart from this consenting issue, sometimes the language also helps, especially when you speak their language* (Kintampo, Ghana)
Informed consent process is well done		- Providing detailed and enough information during the consenting process facilitates disclosure. A good consenting process assures respondents of the use of information gathered to improve their and other’s outcomes. *Sometimes when the respondent has many of such incidence (TOP), when you consent her very well, she will have the mindset that the Ghana Health Service will implement policies that will help to reduce these things and they are willing to give you information* (Kintampo, Ghana)
Feeling justified or open-minded on TOP	- Women are much more open when speaking about miscarriages, though still they hide some information. Abortions or miscarriages are perceived to be related to hereditary or uterine problems (Dabat, Ethiopia)	- Some interviewees disclose TOPs as they feel justified in the reasons for doing it *I found a woman and when I asked that “ have you ever had a miscarriage?” she said that “not even miscarriage but I just aborted” and that the reason was that the rate of conception was high because when the child was only 3 months she could conceive and when she tried family planning she said that “ it was like I had loosen the tap because the bleeding was too much”* (Iganga-Mayuge, Uganda) - Some interviewees do not hide their experiences with TOP, they speak openly *I will say it is the personality, some people are outspoken, they are able to tell what they have gone through, sometimes I myself I get surprised when the women tell me that I aborted this pregnancy despite the difficulty in saying it, sometimes some people come out clear on that* (Kintampo, Ghana)
A sense of regret over TOP	- Some disclose due to guilt they feel over their actions *They talk about it because…I have ever seen a woman who aborted and buried the fetus in the anthill. So, she spoilt her future because over time…she wanted to give birth to a child but in vain* (Iganga-Mayuge, Uganda)	
Perception of speaking to a healthcare provider		- Interviewees feel secure speaking to interviewers they perceive to be health workers *Like the technique of her seeing you in the perspective of “musawo”, [doctor] so she does not want to deceive you, ahaa me, musawo the other husband, we separated, with my husband and that’s why I aborted these pregnancies but don’t let him know and the police will accuse me of this illegal abortions and for you accept to be called musawo but again when you say you are not a nurse, she will ask you why you have been asking her about that and she will say I have been talking with a wrong person* (IgangaMayuge, Uganda)

#### Community perceptions and practices on reporting of TOP

There was a perception reported amongst both women and interviewers from most sites that TOP was immoral and inhuman as per cultural and religious values, resulting in a perceived difficulty in reporting these events. Persons who had undertaken TOP were perceived to have taken a life.

*Why did you abort the baby? Why did you kill a life? (Woman, IgangaMayuge, Uganda)*

Perceived shame associated with TOP was mentioned by women and interviewers as contributing to TOPs being kept secret, and hence rarely reported, especially in young females.

*It is shameful to tell people that you have aborted a pregnancy, so it is not possible to easily disclose it (Woman, Kintampo, Ghana)*

Women observed that grieving openly for a TOP was virtually non-existent as such pain was considered self-inflicted. Interviewers noted that livebirths were easier to report than TOP and some reported them as miscarriages instead.

#### Perceived barriers to reporting TOP

Interviewers felt unable to ask questions on TOP apprehensive of legal, traditional, and religious implications—women also confirmed this notion. According to interviewers, they were perceived at times as disrespectful when they attempted probing women about TOP. Interviewers and women observed that stigmatisation of TOP was very commonly reported in three out the five sites, to the extent that those who had engaged in it were, in many cases, referred to as murderers.

*I think the perception the community has about abortion…how they stigmatise those who have done abortions... Sometimes you are seen as a murderer, so someone who has had an abortion would not like to open up (Interviewer, Kintampo, Ghana)*

This could contribute to TOP being less reported as compared to miscarriages, which are perceived as natural. Younger females out of shyness and perceived stigma were also not willing to disclose TOP as per observations of interviewers.

There was a perception amongst interviewers and women that TOP reporting was not comfortable. Furthermore, unlike information on livebirths that is recorded in antenatal care records, documentation on TOP is hard to come by. Interviewers therefore could only depend on reports from persons who have experienced TOPs.

#### Facilitators of TOP reporting

Interviewers perceived good interviewer communication techniques to facilitate TOP reporting. These included speaking the same language (e.g. English or local) as the interviewees and the use of “softer” contextually appropriate expressions other than a direct reference to TOP.

*What makes it easier for us is instead of asking the woman how many times she has had an abortion, you ask her how many times she has ‘taken out her stomach’ (Interviewer, Bandim, Guinea-Bissau)*

With the diversity of reasons for engaging in TOP and its stigmatised nature, interviewers described needing to develop the tact of adapting to each interviewee’s individual needs whilst not being judgmental. The informed consent process was viewed by interviewers as important in enhancing TOP disclosure by assuring respondents of confidentiality and that information gathered would be used to the benefit of the community.

It was observed by interviewers that when interviewees perceived they are speaking to a healthcare professional, they were much more open in disclosing TOP and other adverse pregnancy outcomes, potentially due to a level of trust for the health professional that might not be available for non-health professionals.

Interviewers observed that individual-level factors perceived to facilitate reporting by the women included personality of the respondent, with some outspoken persons much more likely to report TOP; when the woman felt justified in having a TOP, for example when conceiving immediately after a successful pregnancy; or conversely when a woman regretted an event after she was left with no children.

## Discussion

Measuring TOP is methodologically challenging, and the structure and format of the interview itself is believed to be relevant in eliciting disclosure. Based on data from five countries and 34,371 women of reproductive age, we found that responses to the new questions on TOP were high and facilitated TOP disclosure from women who had not reported them with the roster questions. The new questions could therefore help improve TOP measurement. Termination of pregnancy was perceived as stigmatising, and uncomfortable to disclose, being particularly shameful amongst young women. As such, survey teams should prepare the members to eschew judgmental and stigmatising behaviours and be accommodating of interviewees’ specific circumstances.

Completeness of responses to roster TOP questions, and new Menstrual Restoration and TOP questions were generally high, implying that respondents understood them. A key observation was that once women acknowledged a TOP or delayed menstruation, they reported on most of the other questions related to that event.

With the exception of Guinea-Bissau where no prior rates were identified, significant differences were observed between TOP rates reported in the current study and those from previous studies in countries representing the other study sites [12, 24–27]. These differences could be attributed to TOP characteristics at study sites varying from those in the respective countries overall, methodological differences in data collection, and adjustment factors applied. Also, consistent with prior studies [14, 34], the population TOP rates were underestimated by the roster TOP questions considering the additional responses to the new questions on TOP. The roster TOP questions elicited slightly higher TOP rates in Kintampo, Ghana, when compared to the new questions on TOP, with the reverse happening in Matlab,Bangladesh. Dabat, Ethiopia, had low TOP rates for both the roster and new questions on TOP. Except for the place where they were carried out, respondents who had Menstrual Restoration completely disclosed the processes they went through. Menstrual Restoration rates in Kintampo, Ghana, were similar to TOP rates from the roster TOP and new TOP questions. Menstrual Restoration rates in IgangaMayuge, Uganda, were relatively lower than TOP rates from the roster TOP questions. Differences in the observed rates across sites could be due to cultural variations in how respondents relate to TOPs and their perception of Menstrual Restoration.

Shi and colleagues describe the relevance of response times in determining the characteristics of questions used in surveys, their influence on interviewee’s comprehension, and interviewer performance [31]. Our findings show that average response time for completing the roster TOP questions varied across the five HDSS sites. Comparing these sites, the median response time was highest in Matlab, Bangladesh, and least in Bandim, Guinea-Bissau. Mean time was highest in Matlab, Bangladesh, and lowest in Dabat, Ethiopia. Similarly, mean time spent in answering the new TOP questions was highest in Matlab, Bangladesh, and lowest in Dabat, Ethiopia. No previous population-based studies were found with response times on a similar set of TOP questions, and whilst longer response times may be associated with more considered responses, implementing questions with long response times in a standard DHS survey is unlikely to be feasible.

Respondents in the current study brought to light certain community perceptions and practices that influence TOP reporting. Cultural and religious perceptions of TOP as inhuman and immoral influenced its reporting. Associated societal stigma, moral condemnation, and shaming observed in Cameroon, Kenya, and elsewhere [3, 35–37] resulted in women keeping TOP secret. Perceived stigma led to some women in the current study reporting TOP as miscarriage or not report it altogether [21]. Judgmental attitudes like tagging persons with TOP as taking a life, not having a heart, etc. adversely influenced reporting [21, 37]. Women in a Cameroonian study perceived TOP as immoral and criminal but went ahead for socio-economic reasons [35].

Some perceived barriers to reporting TOP emerged in the current study. Perceived and internalised stigma influence reporting or openly discussing TOP [21, 26, 38]. Fear of adverse reactions like harassment and rejection from partners, parents, etc. made women apprehensive of sharing information of intended TOP, whereas others related it to close friends or siblings who ever had abortions [21, 36–39]. These findings are consistent with previous studies where perceptions of TOP as evil, abnormal, only carried out by commercial sex-workers or murder fuelled feelings of stigma in women [21, 36, 40, 41]. Younger women were much more likely to perceive judgement by others or self-judgement and associated complications of perceived stigma [42], making them less likely to report TOP as in the current study. Huntington and colleagues observed that judgmental or stigmatising attitudes adversely influenced TOP reporting than fear of legal or moral implications [43]. Interviewers in the current study on the contrary wanted to avoid TOP questions apprehensive of legal and religious implications. Survey teams therefore require a deep appreciation of cultural, religious, and other contextual factors that influence TOP reporting inculcated into training of members to enhance their competence at appropriately interacting with study participants.

Perceived facilitators of TOP reporting as per the current study were good interviewer skills such as knowledge of contextually appropriate TOP expressions, adapting to each woman’s individual needs and using a common language, similar to findings of a Cameroonian study [35]. Interviewers’ influence on TOP reporting as per a Malawian study cannot be underestimated [20]. Interviewer gender was not perceived as a facilitator for TOP reporting in the current study in contrast with others from Mexico and some African countries [44, 45]. In our study, some women reported TOP out of feelings of regret and justification of their actions. Individuals’ feelings post-TOP varies—studies have reported school-going females and commercial sex-workers were frequently relieved [37, 46] whilst others with outcomes like infertility and trauma may experience religious and legal guilt coupled with jealousy [46]. Adolescents are more willing to discuss their peers’ experience of TOP than their own [18].

Interviewee trust in healthcare professionals and well-performed informed consent procedures were perceived to enhance TOP reporting in the current study. Consenting for the roster and new questions on TOP were the same; hence, differences in responses could be attributed to the type of questions asked. Question framing and sequencing were perceived to facilitate TOP reporting in the current and Huntington and colleagues’ studies [43], possibly contributing to the additional responses in the new questions on TOP. The roster TOP questions being direct in nature could have generated perceptions of guilt or judgement that made it difficult for respondents to report TOP. The new questions on TOP by their “soft” approach could have made the same respondents feel less judged—possibly resulting in extra TOP reporting.

### Implications of study findings on TOP measurement

Potential next steps for improving direct TOP measurement in women’s questionnaires in population-based surveys premised on findings of this study include (1) enhanced pre-survey interviewer training sessions, to include training in TOP specific terminology used in the communities and training to address stigma and judgmental attitudes, and the need to accommodate interviewee’s specific circumstances; and (2) improved survey processes, including attention to accurate translation of TOP specific questions and interviewer prompts to include commonly understood lay-terms and ensuring the informed consent process is appropriately executed, describing the benefits or otherwise of the study to participants and the wider society.

Specific implications for DHS and Multiple Indicator Cluster Surveys (MICS): The mean and median times for TOP interviews point to the fact that TOP questions would not overly burden respondents or the interview team. Some respondents with a past TOP did not report the event with the standard roster questions, but only with the additional new questions where an additional lead-in question *“It is not uncommon for a woman to get pregnant at a time when circumstance would make it difficult to have a child. Have you ever gotten pregnant at a time when it would have been difficult for you to have a child, or when you did not want to have one?”* was asked prior to the standard question on whether the woman successfully did anything to end that pregnancy. It is possible that this lead-in question may have made women feel more at ease in reporting these events, and the potential for including a similar question in the roster could be considered. A notable observation was that respondents who reported a pregnancy ending in a TOP or Menstrual Restoration went on to respond to further questions regarding that event.

### Strengths and limitations

Strengths of the study include a large and diverse group of study participants from HDSS sites in five countries on the continents of Africa and Asia. A lot of effort went into developing standardised and uniform data collection tools that were tested ahead of the study; this ensured that data generated were comparable across the five study sites and made pooled analysis possible. The Survey Solutions software allowed the assessment of the time women spent in responding to TOP questions, which was a very important aspect of this study. The new questions on Menstrual Restoration and TOP exhibited their potential for eliciting information on women’s management of delayed menstruation and extra TOP events, respectively. Perspectives on factors influencing TOP reporting were enriched by the views of survey interviewers and women who had first-hand experiences related to TOP reporting. We also note that varying legal, religious and cultural differences in the study contexts may have influenced TOP reporting differentially.

### Research gaps for improving measurement of TOP in household surveys

It would be prudent to design and undertake experimental studies powered to ascertain the capacity of the new questions on TOP in eliciting information on TOP in comparison to other questions in population-based surveys. Further exploration of paradata on response time could help determine the characteristics of TOP and menstrual restoration questions that influence interviewee and interviewer performances in such surveys. Except for Bangladesh, Menstrual Regulation/Restoration is rarely reported from the other countries involved in this study; further studies exploring this phenomenon will bridge that knowledge gap.

## Conclusions

All TOP question types were well responded to by the women. The roster TOP questions are likely to under-represent the true population TOP rates. Findings from the current study support further exploration of the new questions using experimental designs in estimating TOP and Menstrual Restoration reporting. During preparations for future population-based surveys, cultural competencies of research teams should be enhanced and the need to adhere to appropriate informed consenting procedures should be emphasised to improve TOP reporting.

## Supplementary Information

**Additional file 1:** Details of qualitative methods (FGDs), EN-INDEPTH study

**Additional file 2:** TOP rates per 1000 women 15–49 years over the five years preceding EN-INDEPTH survey from roster TOP questions.

**Additional file 3:** TOP ratio per 1000 livebirths over the five years preceding EN-INDEPTH survey from roster TOP questions.

**Additional file 4:** Table of EN-INDEPTH survey TOP questions.

**Additional file 5:** STROBE guidelines checklist.

**Additional file 6:** Table of socio-demographic characteristics of focus group discussion participants.

**Additional file 7:** Percentage of women with lifetime and five-year termination of pregnancies from FPH new questions on TOP (three HDSS sites).

**Additional file 8:** Time taken in minutes to complete TOP section by interviewers using paradata.

**Additional file 9:** Ethical approval of local Institutional Review Boards.

## Data Availability

Data sharing and transfer agreements were jointly developed and signed by all collaborating partners. The datasets generated during the current study are deposited online at 10.17037/DATA.00001556 with data access subject to approval by collaborating parties.

## Abbreviations

DHS: Demographic and Health Survey
ENAP: Every Newborn Action Plan
EN-INDEPTH: Every Newborn-International Network for the Demographic Evaluation of Populations & their Health
FBH+: Full Birth History (+ denotes additional questions on pregnancy losses)
FGD(s): Focus group discussion(s)
FIGO: International Federation of Gynaecology and Obstetrics
FPH: Full Pregnancy History
HDSS: Health and Demographic Surveillance System
HICs: High-income countries
LMICs: Low- and middle-income countries
MICS: Multiple Indicator Cluster Surveys
MNCH: Maternal, Newborn and Child Health
SDG: Sustainable Development Goals
TOP: Termination of pregnancy
UN: United Nations
WHO: World Health Organization

## References

[1] Guttmacher Institute (2019). Induced abortion worldwide – global incidence and trends.

[2] Sedgh G, Bearak J, Singh S, Bankole A, Popinchalk A, Ganatra B, Rossier C, Gerdts C, Tunçalp Ö, Johnson BR (2016). Abortion incidence between 1990 and 2014: global, regional, and subregional levels and trends. Lancet.

[3] Singh S, Remez L, Sedgh G, Kwok L, Onda T. Abortion worldwide 2017: uneven progress and unequal access; 2018.

[4] Ganatra B, Gerdts C, Rossier C, Johnson BR, Tunçalp Ö, Assifi A, Sedgh G, Singh S, Bankole A, Popinchalk A (2017). Global, regional, and subregional classification of abortions by safety, 2010–14: estimates from a Bayesian hierarchical model. Lancet.

[5] Ǻhman E, Shah IH (2011). New estimates and trends regarding unsafe abortion mortality. Int J Gynecol Obstet.

[6] Banerjee SK, Andersen KL, Buchanan RM, Warvadekar J (2012). Woman-centered research on access to safe abortion services and implications for behavioral change communication interventions: a cross-sectional study of women in Bihar and Jharkhand, India. BMC Public Health.

[7] Rossier C (2003). Estimating induced abortion rates: a review. Stud Fam Plan.

[8] Salomonsen EL. Unsafe abortion in legally restricted areas. How politics and abortion laws decides women’s future. A literature review on the incidence of induced abortion and adverse health consequences in Sub-Saharan African countries with restrictive abortion laws: UiT Norges arktiske universitet; 2017.

[9] Moreau C, Bajos N, Bouyer J (2004). Question comprehension and recall: the reporting of induced abortions in quantitative surveys on the general population. Population.

[10] Lindberg L, Kost K, Maddow-Zimet I, Desai S, Zolna M. Abortion reporting in the United States: an assessment of three national fertility surveys. Demography. 2020;57:899–925. 10.1007/s13524-020-00886-4 PMC7329789 32458318

[11] Ghana Statistical Service (GSS) GHSG, ICF (2018). Ghana maternal health survey 2017.

[12] Keogh SC, Otupiri E, Chiu DW, Polis CB, Hussain R, Bell SO, Nakua EK, Larsen-Reindorf R (2020). Estimating the incidence of abortion: a comparison of five approaches in Ghana. BMJ Glob Health.

[13] Bell SO, Sheehy G, Hyacinthe AK, Guiella G, Moreau C (2020). Induced abortion incidence and safety in Côte d’Ivoire. PloS one.

[14] Sedgh G, Keogh SC (2019). Novel approaches to estimating abortion incidence. Reprod Health.

[15] Baschieri A, Gordeev VS, Akuze J, Kwesiga D, Blencowe H, Cousens S, Waiswa P, Fisker AB, Thysen SM, Rodrigues A, et al. “Every Newborn-INDEPTH”(EN-INDEPTH) study protocol for a randomised comparison of household survey modules for measuring stillbirths and neonatal deaths in five Health and Demographic Surveillance sites. J Glob Health. 2019;9(1):010901. 10.7189/jogh.09.010901 PMC6377797 30820319

[16] Sánchez-Páez DA, Ortega JA. Reported patterns of pregnancy termination from demographic and health surveys. PLoS One. 2019;14(8):e0221178. 10.1371/journal.pone.0221178 PMC6699730 31425531

[17] Moscrop A (2013). ‘Miscarriage or abortion?’Understanding the medical language of pregnancy loss in Britain; a historical perspective. Med Human.

[18] Singh S, Remez L, Tartaglione A (2010). Methodologies for estimating abortion incidence and abortion-related morbidity: a review.

[19] MacQuarrie KLD, Winfrey W, Meijer-Irons J, Morse A. Consistency of reporting of terminated pregnancies in DHS calendars. In: DHS methodological reports. Rockville: ICF. p. 126.

[20] Leone T, Sochas L, Coast E. Depends who’s asking: interviewer effect on abortion data in sub-Saharan African demographic and health surveys (DHS). In: Population Association of America Annual Meeting; 10-13 April 2019; Austin, Texas; 2018.

[21] Shellenberg KM, Moore AM, Bankole A, Juarez F, Omideyi AK, Palomino N, Sathar Z, Singh S, Tsui AO (2011). Social stigma and disclosure about induced abortion: results from an exploratory study. Global Public Health.

[22] von Elm E, Altman DG, Egger M, Pocock SJ, Gøtzsche PC, Vandenbroucke JP: Strengthening the reporting of observational studies in epidemiology (STROBE) statement: guidelines for reporting observational studies. BMJ. 2007;335:806. 10.1136/bmj.39335.541782.AD PMC2034723 17947786

[23] Guttmacher Institute (2018). Abortion in Africa.

[24] Singh S, Hossain A, Maddow-Zimet I, Vlassoff M, Bhuiyan HU, Ingerick M (2017). The incidence of menstrual regulation procedures and abortion in Bangladesh, 2014. Int Perspect Sex Reprod Health.

[25] Rana J, Sen KK, Sultana T, Hossain MB, Islam RM (2019). Prevalence and determinants of menstrual regulation among ever-married women in Bangladesh: evidence from a national survey. Reprod Health.

[26] Moore AM, Gebrehiwot Y, Fetters T, Wado YD, Bankole A, Singh S, Gebreselassie H, Getachew Y (2016). The estimated incidence of induced abortion in Ethiopia, 2014: changes in the provision of services since 2008. Int Perspect Sex Reprod Health.

[27] Prada E, Atuyambe LM, Blades NM, Bukenya JN, Orach CG, Bankole A (2016). Incidence of induced abortion in Uganda, 2013: new estimates since 2003. PLoS One.

[28] Kwesiga D, Tawiah C, Imam A, Kebede A, Nareeba T, Enuameh YAK, Manu G, Beedle A, Fisker A, Waiswa P, et al. Barriers and enablers to reporting of pregnancy and adverse pregnancy outcomes in population-based surveys: EN-INDEPTH study. BMC Population Health Metrics. 2021;19 (Supplement 1). 10.1186/s12963-020-00228-x PMC7869448 33557858

[29] World Bank (2017). Survey solutions CAPI/CAWI platform: Release 5.26.

[30] Akuze J, Blencowe H, Waiswa P, Baschieri A, Gordeev VS, Kwesiga D, Fisker AB, Thysen SM, Rodrigues A, Biks GA, et al. Randomised comparison of two household survey modules for measuring stillbirths and neonatal deaths in five countries: the Every Newborn-INDEPTH study. Lancet Glob Health. 2020;**8**:e555–66. 10.1016/S2214-109X(20)30044-9 32199123

[31] Shi Y, Feng J, Luo X (2018). Improving surveys with paradata: analytic uses of response time. China Popul Dev Stud.

[32] StataCorp (2017). Stata statistical software: release 15.

[33] QSR International (1999). NVivo qualitative data analysis software.

[34] Bearak JM, Popinchalk A, Sedgh G, Ganatra B, Moller A-B, Tunçalp Ö, Alkema L (2019). Pregnancies, abortions, and pregnancy intentions: a protocol for modeling and reporting global, regional and country estimates. Reprod Health.

[35] Schuster S (2005). Abortion in the moral world of the Cameroon grassfields. Reprod Health Matters.

[36] Ushie BA, Juma K, Kimemia G, Ouedraogo R, Bangha M, Mutua M (2019). Community perception of abortion, women who abort and abortifacients in Kisumu and Nairobi counties, Kenya. PLoS One.

[37] Sorhaindo AM, Juárez-Ramírez C, Olavarrieta CD, Aldaz E, Mejia Pineros MC, Garcia S (2014). Qualitative evidence on abortion stigma from Mexico City and five states in Mexico. Women Health.

[38] Loi UR, Lindgren M, Faxelid E, Oguttu M, Klingberg-Allvin M (2018). Decision-making preceding induced abortion: a qualitative study of women’s experiences in Kisumu, Kenya. Reprod Health.

[39] Kimport K, Foster K, Weitz TA (2011). Social sources of women’s emotional difficulty after abortion: lessons from women’s abortion narratives. Perspect Sex Reprod Health.

[40] Atere AA, Ayodele JO, Omololu O (2012). Abortion and Challenges of Teenage Pregnancy in Lagos, Nigeria. Int J Sci Eng Res.

[41] Cockrill K, Nack A (2013). “I’m not that type of person”: managing the stigma of having an abortion. Deviant Behav.

[42] Makleff S, Labandera A, Chiribao F, Friedman J, Cardenas R, Sa E, Baum SE (2019). Experience obtaining legal abortion in Uruguay: knowledge, attitudes, and stigma among abortion clients. BMC Womens Health.

[43] Huntington D, Mensch B, Miller VC (1996). Survey questions for the measurement of induced abortion. Stud Fam Plan.

[44] Flores-Macias F, Lawson C (2008). Effects of interviewer gender on survey responses: Findings from a household survey in Mexico. Int J Public Opin Res.

[45] Haber N, Robyn PJ, Hamadou S, Yama G, Hien H, Louvouezo D, Fink G. Surveyor gender modifies average survey responses: evidence from household surveys in four Sub-Saharan African Countries [pre-print]. arXiv:181001981 2018.

[46] Oye-Adeniran BA, Adewole IF, Umoh AV, Iwere N, Gbadegesin A (2005). Induced abortion in Nigeria: findings from focus group discussion. Afr J Reprod Health.

