# Supplementary material for: Termination of pregnancy data completeness and feasibility in population-based surveys: EN-INDEPTH study
Source: Popul Health Metr. 2021 Feb 8;19(Suppl 1):12. doi: 10.1186/s12963-020-00238-9 (PMC7869447; doi:10.1186/s12963-020-00238-9)
Supplement: Supplementary file 1 — Additional file 1: Details of qualitative methods (FGDs), EN-INDEPTH study [file 12963_2020_238_MOESM1_ESM.docx]

## Additional file 1: Details of qualitative methods (FGDs), EN-INDEPTH study

Twenty-eight (28) Focus Group Discussions (FGDs) were conducted with 82 EN-INDEPTH survey interviewers and supervisors and 172 women between February and August 2018. The women were sampled purposively from the among those that participated in the EN-INDEPTH survey. Factors considered in the selection of the women included their age, place of residence (rural/urban), and their having experienced an adverse pregnancy outcome including TOPs. The interviewers were sampled purposively from among those who were engaged in the EN-INDEPTH survey consisting of females and males where possible. The number of FGDs undertaken was similar across the sites.

The FGDs explored women’s (respondents) and survey interviewers’ experiences with the EN-INDEPTH survey data collection process, as well as attitudes, knowledge, and practices around reporting and disclosure of pregnancy and adverse pregnancy outcomes and associated enablers and challenges.

A common training and implementation manual was developed for use across the sites. Interviews were held in the most spoken local language (or English) by moderators and note takers who had skills in qualitative data collection methods. Data were transcribed locally and analyzed by HDSS study teams, with at least two analysts participating in coding.
