# Supplementary material for: Termination of pregnancy data completeness and feasibility in population-based surveys: EN-INDEPTH study
Source: Popul Health Metr. 2021 Feb 8;19(Suppl 1):12. doi: 10.1186/s12963-020-00238-9 (PMC7869447; doi:10.1186/s12963-020-00238-9)
Supplement: Supplementary file 2 — Additional file 2: TOP rates per 1000 women 15–49 years over the five years preceding EN-INDEPTH survey from roster TOP questions. [file 12963_2020_238_MOESM2_ESM.docx]

## Additional file 2: TOP rates per 1000 women 15–49 years over the five years preceding EN-INDEPTH survey from FPH roster TOP questions

|  |  | **2012** | **2013** | **2014** | **2015** | **2016** | **2017** | **TOTAL** |
| --- | --- | --- | --- | --- | --- | --- | --- | --- |
| **HDSS** | **N** | **^a^ Rate(95% CI)** | | | | | | |
| Bandim | 4,660 | 1.7(0.7-3.4) | 1.9(0.9-3.7) | 1.5(0.6-3.1) | 1.7(0.7-3.4) | 1.7(0.7-3.4) | 1.3(0.5-2.8) | 10.1(7.4-13.4) |
| Dabat | 6,266 | 0.2(0.0-0.9) | 0.0(0.0-0.5)* | 0.0(0.0-0.5)* | 0.0(0.0-0.5)* | 0.2(0.0-0.9) | 0.0(0.0-0.5)* | 0.3(0.0-1.2) |
| IgangaMayuge | 6,649 | 0.6(0.2-1.5) | 0.2(0.0-0.8) | 0.3(0.0-1.1) | 1.8(0.9-3.2) | 1.2(0.5-2.4) | 1.7(0.8-3.0) | 6.2(4.4-8.4) |
| Kintampo | 6,142 | 3.3(2.0-5.0) | 3.6(2.2-5.4) | 2.8(1.6-4.4) | 2.8(1.6-4.4) | 3.7(2.4-5.6) | 2.4(1.4-4.0) | 19.3(15.9-23.0) |
| Matlab | 10,653 | 1.1(0.6-2.0) | 2.0(1.2-3.0) | 1.2(0.6-2.1) | 2.1(1.3-3.1) | 1.4(0.8-2.3) | 1.6(0.9-2.6) | 9.4(7.6-11.4) |
| Total | 34,371 | 1.3 (1.0-1.8) | 1.5(1.1-2.0) | 1.1(0.8-1.6) | 1.7(1.3-2.2) | 1.6(1.2-2.1) | 1.4(1.1-1.9) | 9.0(8.0-10.0) |
| N= Number of women 15-49; **^a^** Rate = (number of abortions/N) * 1000; CI = Confidence Interval; *one-sided, 95% Cl | | | | | | | |  |
