# Supplementary material for: Termination of pregnancy data completeness and feasibility in population-based surveys: EN-INDEPTH study
Source: Popul Health Metr. 2021 Feb 8;19(Suppl 1):12. doi: 10.1186/s12963-020-00238-9 (PMC7869447; doi:10.1186/s12963-020-00238-9)
Supplement: Supplementary file 3 — Additional file 3: TOP ratio per 1000 livebirths over the five years preceding EN-INDEPTH survey from roster TOP questions. [file 12963_2020_238_MOESM3_ESM.docx]

## Additional file 3: TOP ratio per 1,000 livebirths over the five years preceding EN-INDEPTH survey from FPH roster TOP questions

|  | **2012** | **2013** | **2014** | **2015** | **2016** | **2017** | **TOTAL** |
| --- | --- | --- | --- | --- | --- | --- | --- |
| **HDSS sites** | **TOP Ratio(95% CI) ^a^** | | | | | | |
| Bandim | 9.6(4.2-18.9) | 8.3(3.8-15.7) | 6.3(2.5-12.9) | 6.8(3.0-13.4) | 6.9(3.0-13.5) | 5.5(2.0-12.0) | 7.2(5.3-9.6) |
| Dabat | 1.2(0.0-6.8) | 0.0(0.0 - 3.8)* | 0.0(0.0-3.7)* | 0.0(0.0-3.6)* | 1.3(0.0-7.1) | 0.0(0.0 - 3.4)* | 0.4(0.0-1.4) |
| IgangaMayuge | 5.2(1.4 - 13.1) | 1.2(0.0-6.5) | 2.4(0.3-8.8) | 14.5(7.5-25.2) | 10.1(4.4-19.8) | 13.2(6.6-23.4) | 8.1(5.8-11.0) |
| Kintampo | 13.0(8.5-21.4) | 12.9(8.1-19.5) | 10.1(5.9-16.2) | 10.1(5.9-16.1) | 14.7(9.4-22.0) | 11.7(6.5-19.2) | 12.5(10.3-14.9) |
| Matlab | 7.3(3.8-12.7) | 10.0(6.2-15.3) | 6.4(3.4-11.0) | 10.3(6.4-15.5) | 6.7(3.8-11.1) | 9.9(5.8-15.8) | 8.4(6.9-10.2) |
| Total | 8.2(6.0-10.9) | 7.8(5.8-10.3) | 6.1(4.3-8.3) | 8.9(6.8-11.4) | 8.4(6.4-11.0) | 8.5(6.3-11.2) | 8.1(7.2-9.0) |
| **^a^** Ratio = (number of abortions/livebirths [N]) * 1000; Confidence Interval (CI); *one-sided, 95% Cl | | | | | | | |
