# Supplementary material for: Termination of pregnancy data completeness and feasibility in population-based surveys: EN-INDEPTH study
Source: Popul Health Metr. 2021 Feb 8;19(Suppl 1):12. doi: 10.1186/s12963-020-00238-9 (PMC7869447; doi:10.1186/s12963-020-00238-9)
Supplement: Supplementary file 4 — Additional file 4: Table of EN-INDEPTH survey TOP questions. [file 12963_2020_238_MOESM4_ESM.docx]

## Additional file 4: Table of EN-INDEPTH FPH survey TOP questions

| **Question** | **Responses** | **Additional comments** |
| --- | --- | --- |
| **Roster TOP Questions^1^** | | |
| Roster question (RQ) 1: Was the “pregnancy number”^2^ born alive, born dead, or lost before birth? (alive, dead, lost before term) | Responses were to know whether each pregnancy reported on was born “alive, dead or lost before term” | This question assessed in detail the exact pregnancy outcomes of respondents in the FPH arm of the survey. |
| RQ2: Did you or someone else do something to end this pregnancy?’ (Yes/No) | Responses were “YES/NO” as to whether pregnancies that were not born live were tampered with or not | This question was predicated on RQ1 – inquiring if pregnancies that were reported as “dead or lost before term” had been tampered with |
| **New questions on Menstrual Restoration in the REPRODUCTION SECTION** | | |
| Additional Question (AQ) 1: Have you ever had a period that was more than one week late? (Yes/No) | Responses were “YES/NO” | For women in FPH arm from Dabat, Iganga-Mayuge and Kintampo sites.  Eliciting information as to whether there had been any delays in menstruation – a sign of pregnancy |
| AQ2: Did you do anything to resume your period? | Responses were “YES/NO” | For women in FPH arm from Dabat, Iganga-Mayuge and Kintampo sites.  Eliciting information as to whether the delay in AQ1 was resolved by some external influence |
| AQ3: What did you do to resume your period?’ | Responses were “Pill/Inj/Herb/Other” | For women in the FPH arm from Dabat, Iganga-Mayuge and Kintampo sites who responded “YES” to AQ2. This question was to determine the approach used for pregnancy termination |
| AQ4: Where did you go to get help to get your period back? | Response was a “location” | For women in the FPH arm from Dabat, Iganga-Mayuge and Kintampo sites who responded “YES” to AQ2. The question was to identify “Where” persons who terminated a pregnancy went to for help |
| **New questions on TOP** | | |
| New Questions (NQ) 1: Women sometimes have pregnancies that do not result in a live born child. That is, a pregnancy can end in a miscarriage, abortion or the child can be born dead. Have you ever had a pregnancy that did not end in a live birth? 5 sites (yes/no) | Response was “Yes/No” | Asked for all women from the FPH arm from all five sites.  Responses were to determine whether some pregnancies ended up not in live births. |
| NQ2: It is not uncommon for a woman to get pregnant at a time when circumstance would make it difficult to have a child. Have you ever gotten pregnant at a time when it would have been difficult for you to have a child, or when you did not want to have one?’ 4 sites (Yes/No) | Responses were “Yes/No” and predicated on NQ1. | Asked for all women from the FPH arm from three sites i.e. Dabat, Kintampo and Matlab who did not report a TOP in the pregnancy roster question. |
| NQ3: Did you or anyone else ever successfully do anything to end that pregnancy? [abortion over lifetime] | Responses were “Yes/No” to whether abortion was ever done and predicated on NQ2 | Responses were from women of the FPH arm in Dabat, Kintampo and Matlab who did not report a TOP in pregnancy roster section. |
| NQ4: Did you have such a pregnancy in the last five years? (Yes/No) | Response was “Yes/No” to whether abortion was done in the five years preceding the EN INDEPTH survey | Asked of all women from the FPH arm from Dabat, Kintampo and Matlab who did not report a TOP in the pregnancy roster question. |
| NQ5: What was the MAIN reason you decided to have this (last) abortion? | Response was for reason of abortion | Asked to all women from the FPH arm in Dabat, Kintampo and Matlab sites reporting a TOP in the last 5 years in response to NQ4. |
| NQ6: What did you do to end this pregnancy? | Response was on approach used in aborting pregnancy | Asked to all women from the FPH arm in Dabat, Kintampo and Matlab sites reporting a TOP in the last 5 years in response to NQ4. |
| NQ7: Who suggested that you might have an abortion?’ | Response was on who advised the performance of the abortion. | Asked to all women from the FPH arm in Dabat, Kintampo and Matlab sites reporting a TOP in the last 5 years in response to NQ4. |
| NQ8: Who did you see to get this done?’ | Who performed the abortion (person who performed the abortion) | Asked to all women from the FPH arm in Dabat, Kintampo and Matlab sites reporting a TOP in the last 5 years in response to NQ4. |
| NQ9: Where did you go to get this done?’ | Where abortion was done (location of abortion) | Asked to all women from the FPH arm in Dabat, Kintampo and Matlab sites reporting a TOP in the last 5 years in response to NQ4. |
| NQ10: How much did you pay for this abortion, including gifts or money given to the doctor (or person who performed this abortion)?’ | Cost of abortion | Asked to all women from the FPH arm in Dabat, Kintampo and Matlab sites reporting a TOP in the last 5 years in response to NQ4. |

^1^ Standard wording used in FPH in Nepal 2016 DHS. ^2^ Where is given by the respondent for the child the name is used. Where no name is given the interviewers used the pregnancy number.
