# Supplementary material for: Termination of pregnancy data completeness and feasibility in population-based surveys: EN-INDEPTH study
Source: Popul Health Metr. 2021 Feb 8;19(Suppl 1):12. doi: 10.1186/s12963-020-00238-9 (PMC7869447; doi:10.1186/s12963-020-00238-9)
Supplement: Supplementary file 6 — Additional file 6: Table of socio-demographic characteristics of focus group discussion participants. [file 12963_2020_238_MOESM6_ESM.docx]

#

## Additional file 6: Table of socio-demographic characteristics of focus group discussion participants

| **Characteristics of participants of Focus Group Discussions** | | | | | | | | | | | | |
| --- | --- | --- | --- | --- | --- | --- | --- | --- | --- | --- | --- | --- |
|  | **Bandim** | | **Dabat** | | **IgangaMayuge** | | **Kintampo** | | **Matlab** | | **Total** | |
|  | **Women** | **Interviewers** | **Women** | **Interviewers** | **Women** | **Interviewers** | **Women** | **interviewers** | **Women** | **Interviewers** | **Women** | **Interviewers** |
| No. of FGDs | 4 | 1 | 3 | 1 | 4 | 2 | 4 | 2 | 4 | 3 | 19 | 9 |
| **Age of respondents in years** | | | | | | | | | | | | |
| < 25 | 8 | 6 | 0 | 8 | 15 | 0 | 14 | 1 | 13 | 2 | 50 | 17 |
| 25 – 34 | 15 | 3 | 13 | 2 | 11 | 10 | 25 | 13 | 3 | 7 | 67 | 35 |
| 35 – 49 | 6 | 2 | 11 | 0 | 14 | 10 | 10 | 4 | 12 | 13 | 53 | 29 |
| 50 + | 0 | 0 | 0 | 0 | 0 | 0 | 0 | 0 | 0 | 1 | 0 | 1 |
| Missing | 0 | 0 | 1 | 0 | 0 | 0 | 1 | 0 | 0 | 0 | 2 | 0 |
| **Sex of respondents** | | | | | | | | | | | | |
| Female | 29 | 9 | 25 | 7 | 40 | 10 | 50 | 3 | 28 | 15^1^ | 172 | 44 |
| Male | 0 | 2 | 0 | 3 | 0 | 10 | 0 | 15 | 0 | 4^1^ + 4^2^ | 0 | 38 |
| Total | 29 | 11 | 25 | 10 | 40 | 20 | 50 | 18 | 28 | 23 | 172 | 82 |
| ^1^ supervisors; ^2^ data collectors | | | | | | | | | | | | |

## 
