# Supplementary material for: Termination of pregnancy data completeness and feasibility in population-based surveys: EN-INDEPTH study
Source: Popul Health Metr. 2021 Feb 8;19(Suppl 1):12. doi: 10.1186/s12963-020-00238-9 (PMC7869447; doi:10.1186/s12963-020-00238-9)
Supplement: Supplementary file 7 — Additional file 7: Percentage of women with lifetime and five-year termination of pregnancies from FPH new questions on TOP (three HDSS sites). [file 12963_2020_238_MOESM7_ESM.docx]

## Additional file 7: Percentage of women with lifetime and five-year termination of pregnancies from FPH new TOP questions (three HDSS sites)

| **Proportion of women who had lifetime termination of pregnancy** | |
| --- | --- |
| **HDSS sites** | **Percentage(95% CI)** |
| Dabat (N=608) | 2.0(1.0 – 3.4) |
| Matlab(N=1,792) | 15.5(13.9 - 17.3) |
| Kintampo(N=470) | 11.5(8.6 – 14.7) |
| Total (N=2,870) | 12.0(10.8 - 13.2) |
|  |  |
| **Proportion of women who had termination of pregnancy in the last five years preceding the survey** | |
| **HDSS sites** | **Percentage(95% CI)** |
| Dabat (N=12) | 8.3(0.2 – 38.5) |
| Matlab(N=278) | 58.6(52.6 - 64.5) |
| Kintampo(N=54) | 18.5 (9.3 - 31.4) |
| Total (N=344) | 50.6(45.2 – 56.0) |
