# Supplementary material for: Termination of pregnancy data completeness and feasibility in population-based surveys: EN-INDEPTH study
Source: Popul Health Metr. 2021 Feb 8;19(Suppl 1):12. doi: 10.1186/s12963-020-00238-9 (PMC7869447; doi:10.1186/s12963-020-00238-9)
Supplement: Supplementary file 8 — Additional file 8: Time taken in minutes to complete TOP section by interviewers using paradata. [file 12963_2020_238_MOESM8_ESM.docx]

## Additional file 8: Time taken in minutes to complete FPH TOP section by interviewers based on extracted paradata

|  |  | **Mean time±SD*** | **25^th^ Percentile** | **Median** | **75^th^ Percentile** | **Interquartile range** |
| --- | --- | --- | --- | --- | --- | --- |
| **HDSS sites that answered Menstrual restoration questions** | | | | | | |
| Dabat |  | 1.6±2.7 | 0.6 | 0.8 | 1.2 | 0.6 |
| IgangaMayuge |  | 1.1±2.0 | 0.6 | 0.7 | 1.1 | 0.5 |
| Kintampo |  | 1.7±2.7 | 0.6 | 1.0 | 1.8 | 1.1 |
| Overall |  | 1.4±2.5 | 0.6 | 0.8 | 1.3 | 0.7 |
|  |  |  |  |  |  |  |
| **HDSS that answered New questions on TOP** | | | | | | |
| Dabat |  | 1.5±1.9 | 0.6 | 0.8 | 0.9 | 0.4 |
| Matlab |  | 5.0±4.8 | 2.1 | 3.5 | 6.2 | 4.1 |
| Kintampo |  | 2.5±2.2 | 1.3 | 2.2 | 3.0 | 1.8 |
| Overall |  | 4.2±4.3 | 1.6 | 2.7 | 4.9 | 3.3 |
|  |  |  |  |  |  |  |
| **HDSS sites that answered Roster TOP questions** | | | | | | |
| Bandim |  | 1.2±1.1 | 0.6 | 0.7 | 1.6 | 1.0 |
| Dabat |  | 1.1±0.8 | 0.5 | 0.8 | 2.0 | 1.4 |
| IgangaMayuge |  | 2.1±4.5 | 0.6 | 0.8 | 1.1 | 0.5 |
| Matlab |  | 3.7±4.7 | 0.9 | 1.3 | 4.7 | 3.8 |
| Kintampo |  | 2.1±3.0 | 0.6 | 0.8 | 2.1 | 1.5 |
| Overall |  | 2.5±3.8 | 0.6 | 1.0 | 2.4 | 1.7 |

*SD = Standard deviation
